# Distinct esophageal adenocarcinoma molecular subtype has subtype-specific gene expression and mutation patterns

**DOI:** 10.1186/s12864-018-5165-0

**Published:** 2018-10-24

**Authors:** Xiangqian Guo, Yitai Tang, Wan Zhu

**Affiliations:** 1 Department of Preventive Medicine, Joint National Laboratory for Antibody Drug Engineering, Institute of Biomedical Informatics ,School of Basic Medical Sciences, Henan University, Kaifeng, 475004 China; 2 Cell Signal Transduction Laboratory, Henan University, Kaifeng, 475004 China; 3 Department of Pathology, Stanford University School of Medicine, 300 Pasteur Drive, Stanford, CA 94305 USA; 4 Department of Anesthesia, Stanford University, 300 Pasteur Drive, Stanford, CA 94305 USA

**Keywords:** Esophageal adenocarcinoma, Molecular subtype, Gene expression, Mutation, Therapy

## Abstract

**Background:**

Esophageal carcinoma (EC), consists of two histological types, esophageal squamous carcinoma (ESCC) and esophageal adenocarcinoma (EAC). EAC accounted for 10% of EC for centuries; however, the prevalence of EAC has alarmingly risen 6 times and increased to about 50% of EC in recent 30 years in the western countries, while treatment options for EAC patients are still limited. Stratification of molecular subtypes by gene expression profiling methods had offered opportunities for targeted therapies. However, the molecular subtype in EAC has not been defined. Hence, Identification of EAC molecular subtypes is needed and will provide important insights for future new therapies.

**Results:**

We performed meta-analysis of gene expression profiling data on three independent EAC cohorts and showed that there are two common molecular subtypes in EAC. Each of the two EAC molecular subtypes has subtype specific expression patterns and mutation signatures. Genes which were over-expressed in subtype I EACs rather than subtype II EAC cases, were enriched in biological processes including epithelial cell differentiation, keratinocyte differentiation, and KEGG pathways including basal cell carcinoma. *TP53* and *CDKN2A* are significantly mutated in both EAC subtypes. 24 genes including *SMAD4* were found to be only significantly mutated in subtype I EAC cases, while 30 genes including *ARID1A* are only significantly mutated in subtype II EACs.

**Conclusion:**

Two EAC molecular subtypes were defined and validated. This finding may offer new opportunities for targeted therapies.

**Electronic supplementary material:**

The online version of this article (10.1186/s12864-018-5165-0) contains supplementary material, which is available to authorized users.

## Background

Esophageal carcinoma (EC) is the sixth most common cause of cancer death in the world [1]. Based on the 2017 estimates by the American Cancer Society, approximate 16,940 new EC cases (13,360 in men and 3,580 in women) were diagnosed and about 15,690 deaths (12,720 in men and 2,970 in women) were estimated. EC consists of two histological types, esophageal squamous carcinoma (ESCC) and esophageal adenocarcinoma (EAC). Current treatments for both types of EC are similar, including chemotherapy, radiation therapy and surgery whilst surgery is the most common treatment method. Despite the static low proportion (approximate 10%) of EAC in EC in the Asian countries, there is an alarming increase of EAC in western countries in recent 30 years, which makes EAC account for half of all the EC cases and as one of the fastest growing malignancies in the US. Though the survival rate of ESCC patients has been improved in recent years, the death rate is still high for EAC patients, suggesting that more efforts should be placed on studying EAC.

In the past decade, the success in molecular stratification and identification of a number types of tumors, e.g. breast cancer, lung cancer, bladder cancer, colon cancer and leiomyosarcoma, into distinct subtypes lead to significant improvement of our knowledge in these malignancies. More importantly, these new findings have led to discoveries of novel targeted therapeutic approaches for treating these cancers [2–19]. However, little is known about the molecular heterogeneity (subtypes) of EAC and no targeted therapy currently exists for EAC. Therefore, recognition of different molecular subtypes for EAC will not only improve our understanding of the mechanisms underlining tumorigenesis and tumor progress; but the successful identification of EAC molecular subtypes and the diagnostic markers for these subtypes will also lay the foundation for the development of targeted therapies for EAC.

Within this study, we performed meta-expression-profiling analysis in three independent big cohorts of EAC, and demonstrated that there are two distinct molecular subtypes of EAC. Furthermore, each of these subtypes has distinct expression pattern and mutation profile.

## Result

### Consensus clustering identified two reproducible molecular subtypes of esophageal adenocarcinoma

Gene expression profiles of 88, 75 and 52 cases of EAC were collected from three independent cohorts, including TCGA [20], GSE13898 [21] and GSE19417 [22]. Clinicopathological characteristics of each cohort were described in Additional file 1: Table S1.

**Fig. 1:**
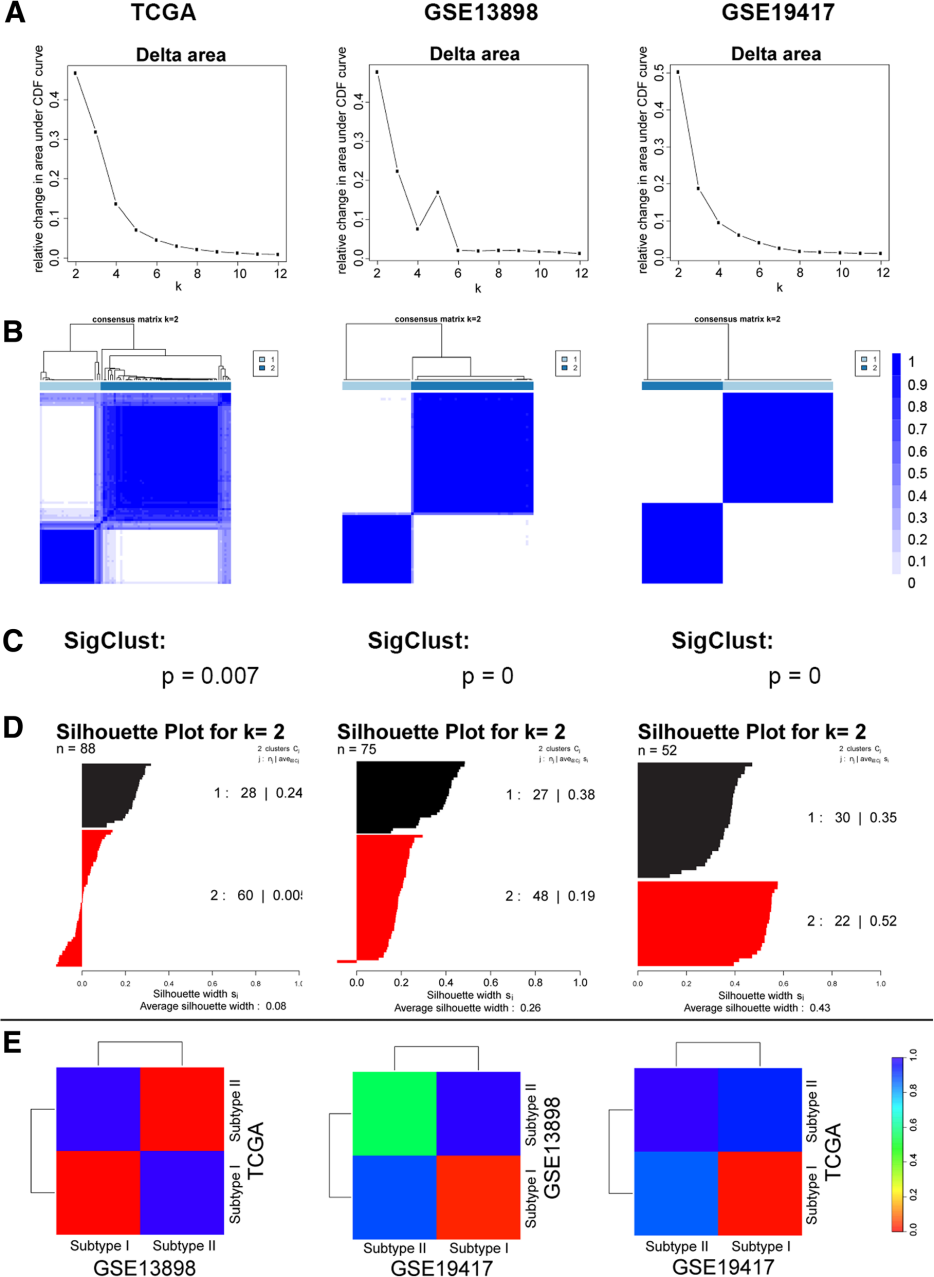
Identification of two molecular subtypes of esophageal adenocarcinoma. **a** Area under empirical cumulative distribution plots (k = 2 to k = 12) determined the two optimal molecular subtypes of EAC. (k denotes the number of clusters). **b** Consensus matrix displaying the two robust subgroups of EAC were defined by gene expression. **c** Significance analysis of subtype classifications in each dataset was determined by SigClust. **d** Silhouette analysis of EAC samples based on the assignments from Consensus Clustering. **e** Subclass association (SA) matrix of molecular subtypes between three cohorts. The rainbow bar indicates the FDR-corrected *p* value

In order to determine the number of subtypes of EAC, consensus clustering was performed on each of the three datasets independently after filtered with standard deviation. Despite distinct profiling methods used by these three independent datasets, we consistently found two robust and stable subtypes in all three datasets (Fig. 1). The ratio of subtype I to subtype II EAC patients was 1:1.5, 1:1.7 and 1:0.7 for dataset TCGA, GSE13898 and GSE19417, respectively (Additional file 1: Table S1). Further analysis using SigClust demonstrated that the two subtypes in each cohort were all statistically significantly different (Fig. 1c). Subsequent Silhouette analysis indicated that 68% (60/88), 99% (74/75) and 100% (52/52) of samples in TCGA, GSE13898 and GSE19417 had positive silhouette values validating the assignments from consensus clustering. The samples with positive silhouette values were defined as “core samples” for subsequent analysis as performed previously [12, 23], unless otherwise stated (Fig. 1d).

To analyze the reproducibility of molecular subtypes between independent cohorts, subclass mapping was performed. Subclass mapping analysis on EAC cases with positive silhouette values showed that subtype I EAC were significantly reproducible among all the cohorts, while subtype II EAC were significantly reproducible in two of the three cohorts (TCGA and GSE13898) (Fig. 1e). The possible reasons for the inconsistent reproducibility of subtype II EAC in GSE19417 cohort might be the disproportionate EAC population in GSE19417 (EAC ratio of two subtypes 1:0.7 in GSE19417 vs.1:1.5/1:1.7 in other two cohorts) and different gene expression profiling methods used.

### Clinical features of esophageal adenocarcinoma molecular subtypes in three datasets

The T-staging (size or direct extent of the primary tumor) was found to be significantly different between two EAC molecular subtypes in GSE13898 (*p* = 0.0009). There is no association between molecular subtypes with differentiation (*p* = 0.9488), grade (*p* = 0.3249 in TCGA, *p* = 0.5345 in GSE13898), alcohol (*p* = 0.8195), smoke (*p* = 0.5569), positive nodes (*p* = 0.2886), or origin of countries (*p* = 0.1326) (Additional file 1: Table S1). In addition, there is no significant difference of overall survivals between the two EAC molecular subtypes in each of the three datasets. These association analyses indicate that the EAC molecular subtypes are relatively independent of current clinical features.

### Identification of biological pathways and processes representative of two molecular subtypes

To identify the differentially expressed genes between two molecular subtypes of EAC, SAMseq analysis on EAC cases with positive silhouette values was performed between subtype I and subtype II EAC using the TCGA dataset. A total of 678 genes were significantly over-expressed in subtype I cases than subtype II cases including gene *NGFR*, *CXCR2* and *ATP6V0A4*, while no genes were identified to be over-expressed in subtype II than in subtype I cases by SAMseq analysis (Additional file 1: Table S2). These 678 genes were enriched in biological processes including epithelial cell differentiation, keratinocyte differentiation (Additional file 1: Table S3), and were enriched in KEGG pathways including basal cell carcinoma (Additional file 1: Table S4).

**Fig. 2:**
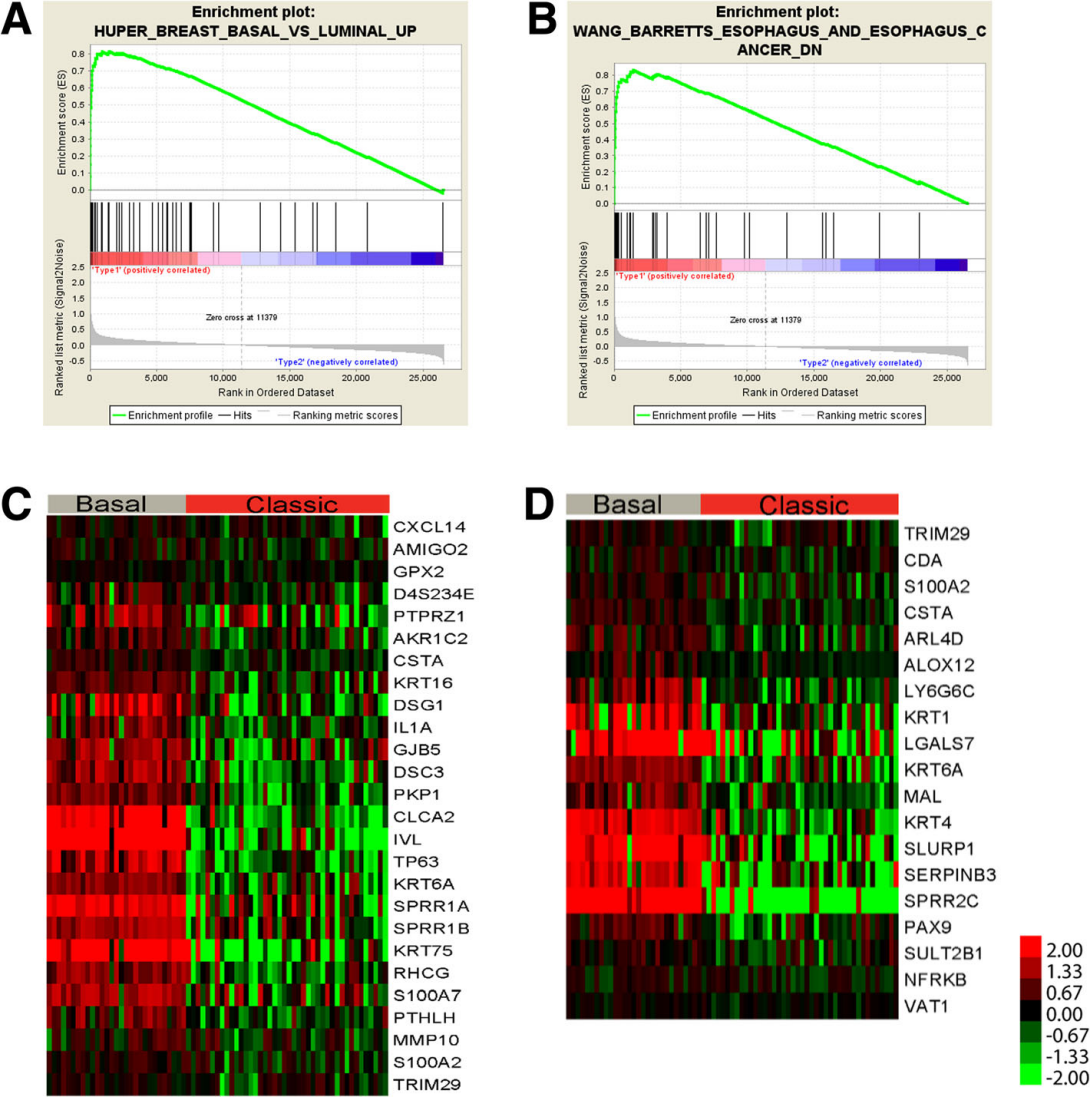
GSEA gene sets and relative expression heatmap. **a**. GSEA Enrichment plot for gene set HUPER_BREAST_BASAL_VS_LUMINAL_UP. **b** GSEA Enrichment plot for gene set WANG_BARRETTS_ESOPHAGUS_AND_ESOPHAGUS_CANCER_DN. **c** Heatmap of core enrichment genes for gene set HUPER_BREAST_BASAL_VS_LUMINAL_UP. **d** Heatmap of core enrichment genes for gene set WANG_BARRETTS_ESOPHAGUS_AND_ESOPHAGUS_CANCER_DN. Note. “Basal” and “Classic” stand for Subtype I and Subtype II EAC, respectively

**Fig. 3:**
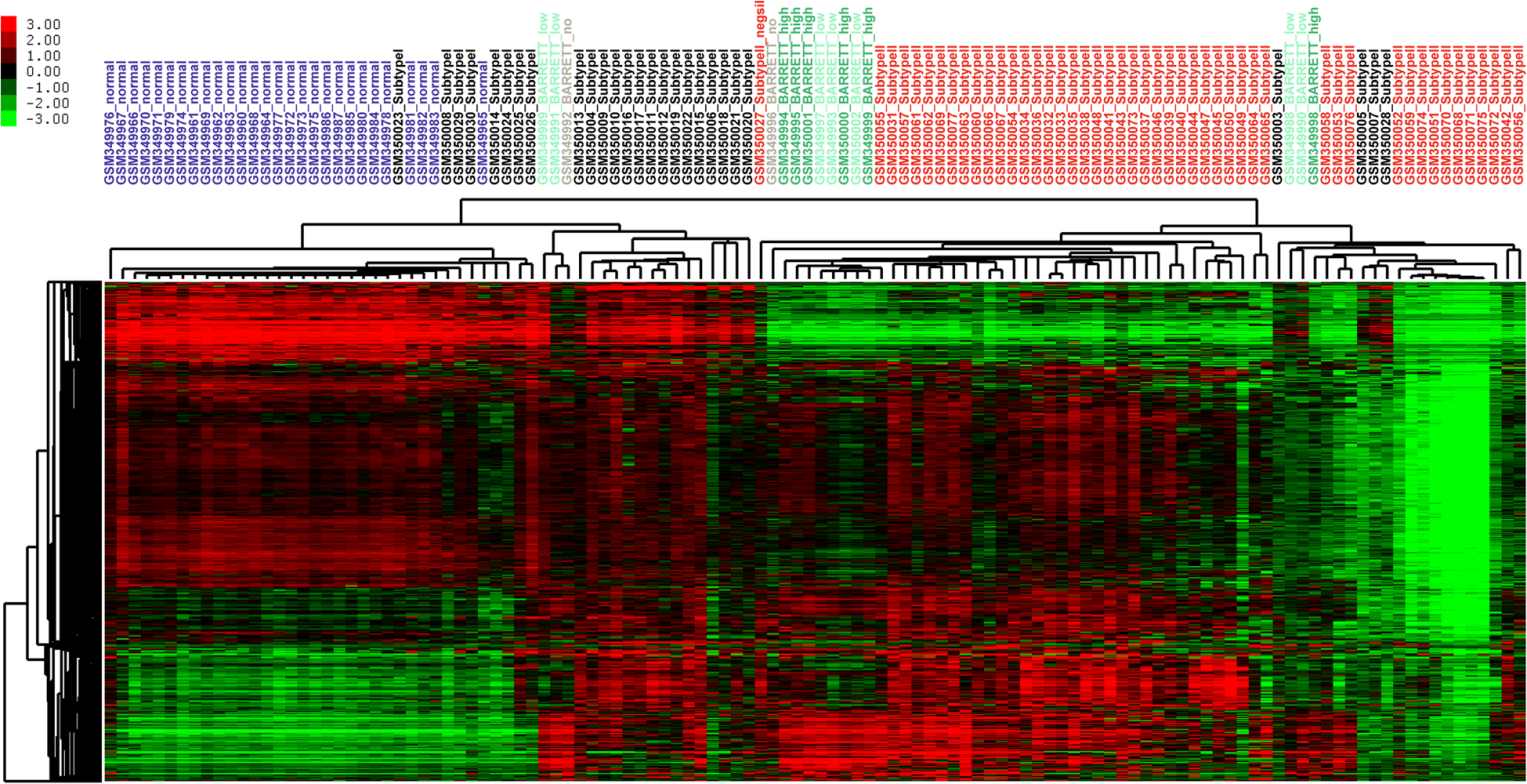
Unsupervised hierarchical clustering of two subtypes of EAC, normal esophagus, and BE. “BARRETT_low” denotes low grade dysplasia in Barrett’s esophagus, while “BARRETT_high” means high grade dysplasia in Barrett’s esophagus. “BARRETT_no” stands for non-dysplastic Barrett’s esophagus (data from GSE13898)

**Fig. 4:**
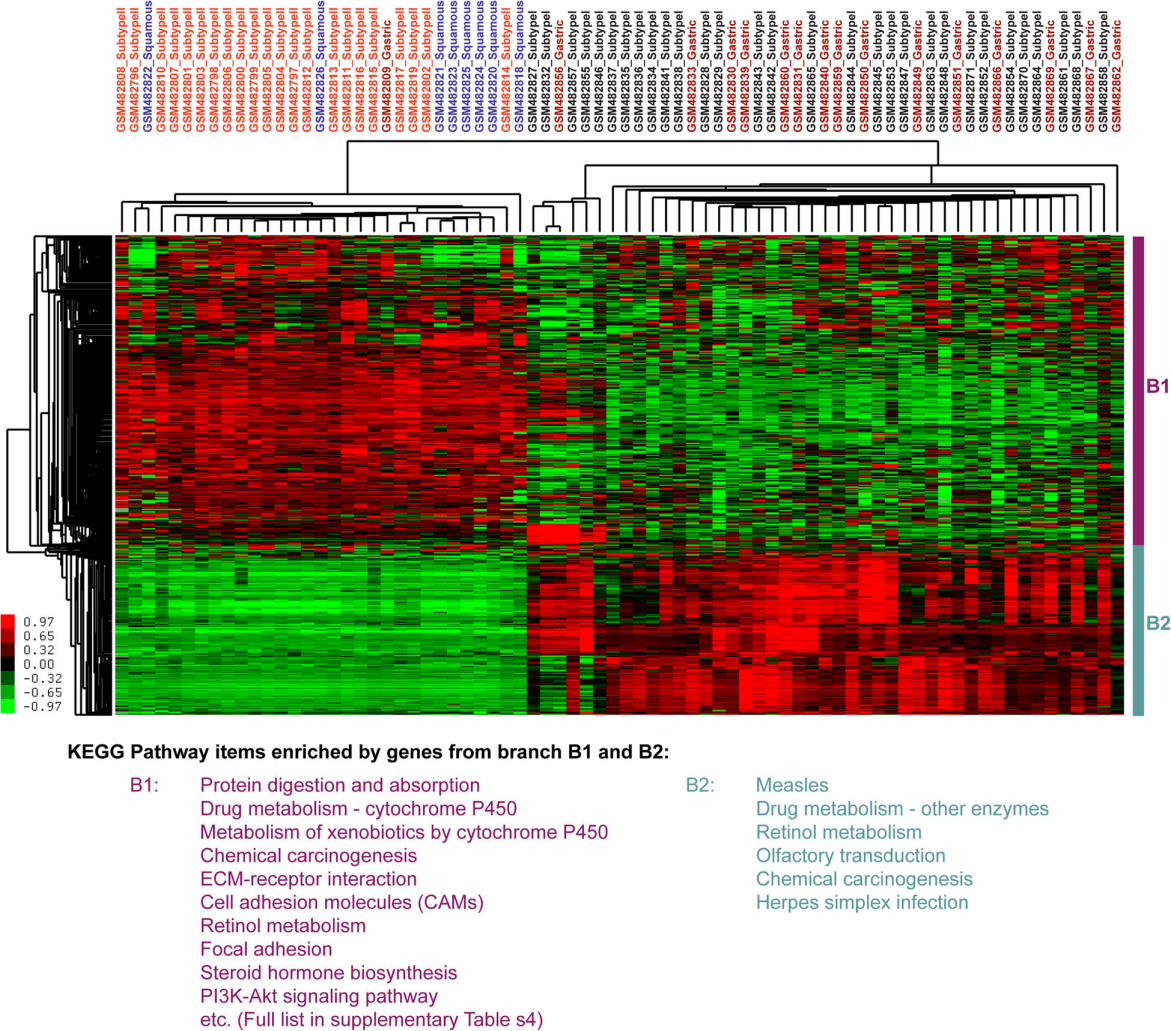
Unsupervised hierarchical clustering of two subtypes of EAC, gastric and squamous carcinoma. “Gastric” labelled above heatmap denotes gastric adenocarcinoma while “Squamous” stands for squamous cell carcinoma (Data from GSE19417)

In addition, Gene Set Enrichment Analysis (GSEA) was performed on EAC cases with positive silhouette values to identify subtype specific biological processes, signaling pathways and potential biomarkers. Subtype I EAC was enriched by curated gene sets including BASAL set (HUPER_BREAST_BASAL_VS_LUMINAL_UP) and WANG set (WANG_BARRETTS_ESOPHAGUS_AND_ESOPHAGUS_CANCER_DN). BASAL set consisted of genes that were up-regulated in basal mammary epithelial cells compared to the luminal ones [24]. WANG set was comprised of genes that were down-regulated in EAC and Barret’s esophagus (BE) relative to normal esophagi (Fig. 2) [25]. The unsupervised hierarchical clustering of normal esophageal tissues, subtype I, subtype II and BE, showed that subtype II shared the most similar expression patterns with BE with the notion that BE was reported to be the precursor of classic EAC while subtype I EAC cases were closest to normal tissues (Fig. 3). When unsupervised hierarchical clustering the cases of two subtypes of EAC, squamous esophageal carcinoma and gastric carcinoma, we found that subtype I EAC cases shared most similar molecular expression profiles with gastric carcinoma while subtype II EAC cases overwhelmingly co-clustered with squamous carcinoma (Fig. 4). In addition, the genes shared by subtype I EAC and gastric cancer were enriched in KEGG pathways including drug metabolism-other enzymes, while KEGG pathways, including Drug metabolism-cytochrome P450 were common for subtype II EAC and squamous carcinoma (Fig. 4 and Additional file 1: Table S4).

The comprehensive study of esophageal carcinoma from TCGA [26] revealed that EAC strongly resembled CIN (Chromosomal Instability) gastric adenocarcinoma. Our current study further divided EAC population into sub-populations, subtype I and subtype II EACs, and showed that subtype I EAC shared more common expression patterns with gastric adenocarcinoma (Fig. 4).

### Esophageal adenocarcinoma subtype-specific somatic mutations

**Fig. 5:**
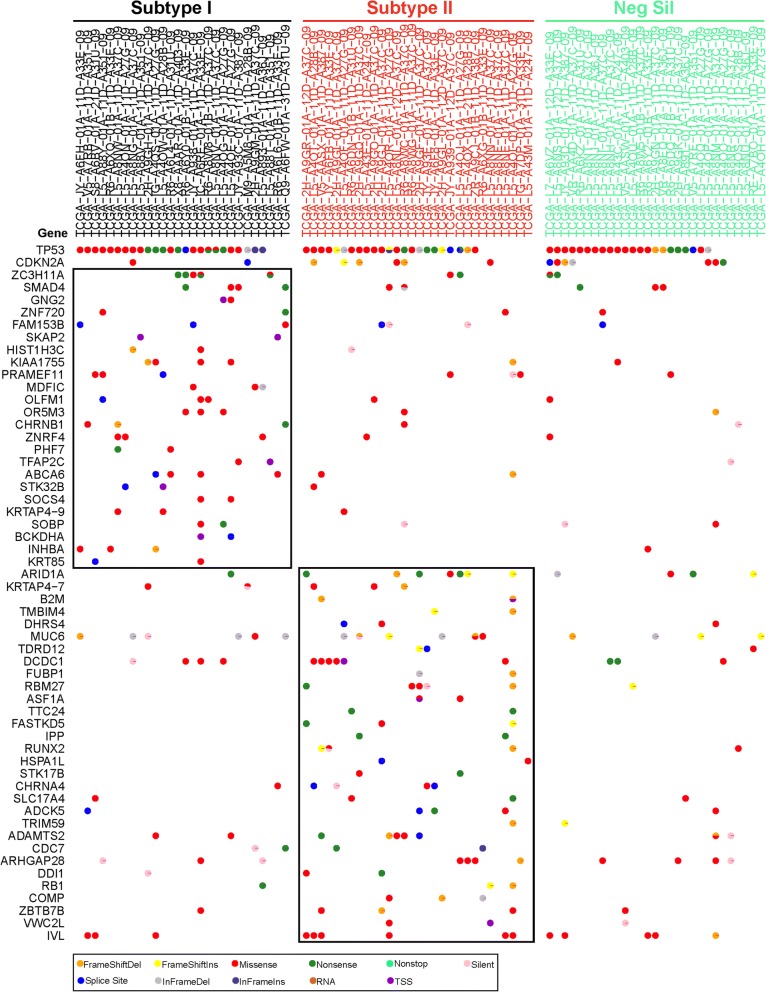
Somatic mutation profiles of EAC across two molecular subtypes

In the TCGA cohort, 87 of 88 esophageal adenocarcinoma profiled by RNA-Seq was also analyzed by exome-Seq, including 28 subtype I cases with positive silhouette values, 30 subtype II cases with positive silhouette values and 29 cases with negative silhouette values. An average of 254, 311 and 279 somatic mutations were identified in subtype I, subtype II cases with positive silhouette values and cases with negative silhouette values, respectively (Fig. 5). No significant difference in the numbers of mutation between subtype I and subtype II was found (Additional file 1: Table S6 and Additional file 2: Figure S1, *p* = 0.5522, T-test).

When analyzed all these 87 EAC cases as a population using MutSigCV analysis, 69 genes, including *TP53*, *CDKN2A*, *MUC6*, *ARID1A*, *ERBB2* and *SMAD4*, were found significantly mutated in this cohort (*p* < 0.01). Four of these 69 genes (*TP53*, *CDKN2A*, *ARID1A* and *SMAD4*) were known somatic mutations in EAC.

To identify subtype specific somatic mutations, MutSigCv analysis was performed on core subtype I and core subtype II cases, respectively. As showed, only *TP53* and *CDKN2A* genes were significantly mutated in both subtype I and subtype II EAC. Twenty-four genes, including *SMAD4*, *SOCS4* and *SKAP2*, were only significantly mutated in subtype I EAC, while 30 genes, including *ARID1A*, *DCDC1* and *IVL*, were only significantly somatically mutated in subtype II cases (Fig. 5). KEGG pathway analysis showed that genes significantly mutated in subtype I EAC (*TP53*, *CDKN2A* and *SMAD4*) and genes significantly mutated in subtype II EAC (*TP53*, *CDKN2A* and *RB1*, *CDC7*) were significantly enriched in pathways including cell cycle.

### Association between molecular subtypes and response to chemotherapy

Studying the association between molecular subtypes and response to therapies will help to guide the future treatment for EAC patients. In the TCGA dataset, excluding patients with negative silhouette values and thus less reliable subtype assignments, only three patients took chemotherapy. Among these three patients, two were from subtype II and both had a complete response to chemotherapy, while one was from subtype I and regrettably had clinical progressive disease, nevertheless the statistic test between subtypes and chemotherapy did not reach significance (Additional file 1: Table S1, *p* = 0.0833). In addition, only one patient with positive silhouette values, who was from subtype II, took radiotherapy and had a complete response.

To further investigate the genes relevant to chemo-sensitivity/resistance, we compared the gene expression profiles between four EAC patients with complete responses to chemotherapy and three patients with progressive diseases from the TCGA data by SAMseq analysis (including the cases with negative silhouette values). As a result, 219 genes were significantly over-expressed in patients with progressive diseases while no gene was over-expressed in patients with complete responses to chemotherapy. Forty-five of these 219 genes were also over-expressed in subtype I compared to subtype II EAC. These overlapped genes include *ATP6V0A4*, *BMP7*, *KLK11*, etc. (Additional file 1: Table S5), and were enriched in GO biological processes including proteolysis, epithelial cell differentiation and epithelium development.

### Identification of biomarkers for esophageal adenocarcinoma subtypes

**Fig. 6:**
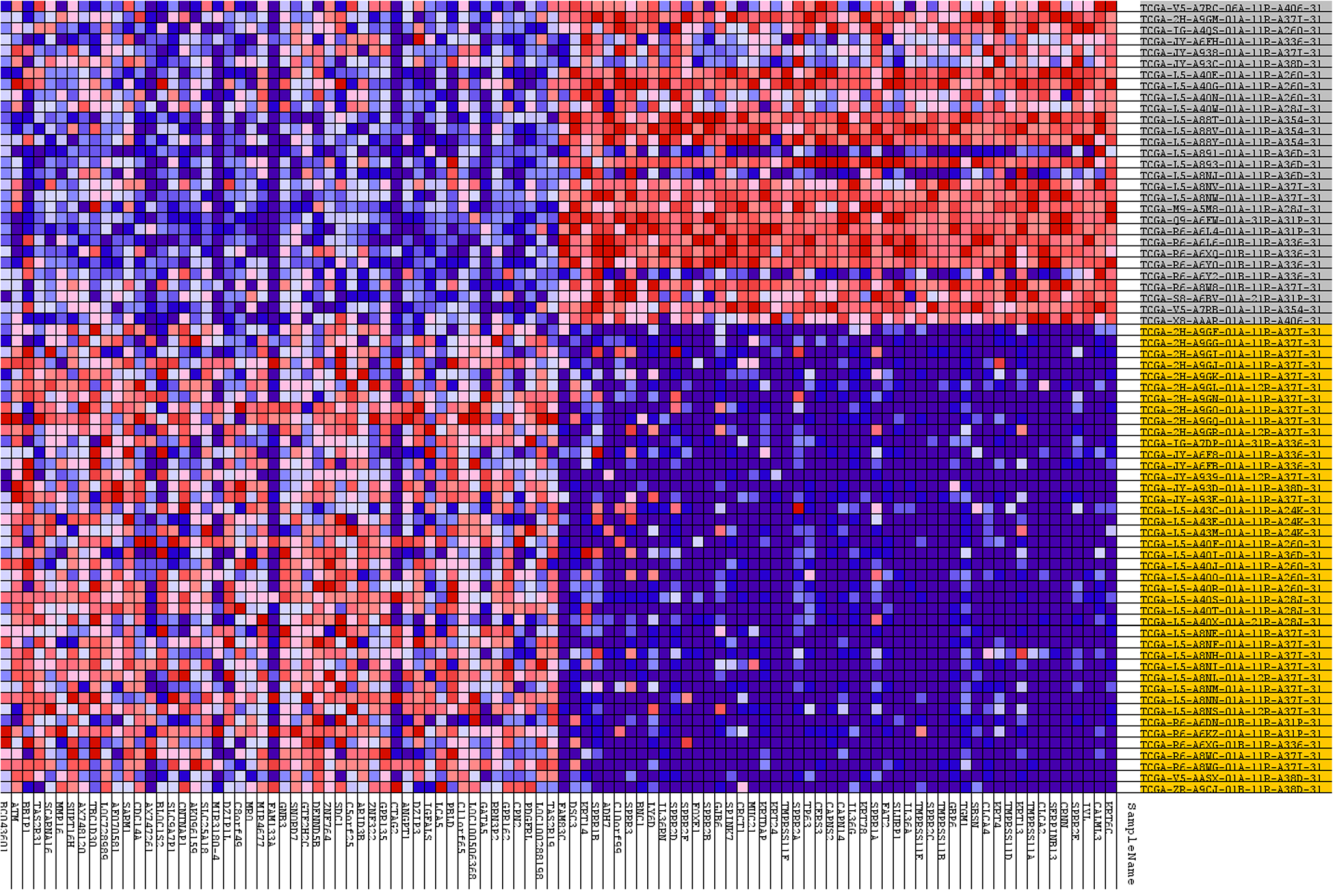
Heatmap of top50 genes from subtype I and subtype II EAC

Identification of biomarkers for molecular subtypes of EAC will provide new insights to the future diagnosis of these subtypes and guide the subtype-specific and –effective therapies. GSEA and SAMseq analyses were used to identify potential markers for these two molecular subtypes. GSEA identified top 50 genes as potential biomarkers for each subtype while 678 genes were shown to be significantly over-expressed in subtype I than subtype II cases by SAMseq analysis. The comparison between GSEA and SAMseq found that all the top 50 genes from GSEA analysis for subtype I were also significantly over-expressed in subtype I than subtype II by SAMseq analysis (Fig. 6).

Among the top 50 genes, *TP63* is a transcription factor from p53 family and was found to be significantly over-expressed in subtype I than subtype II EAC. The additional molecular test (from GSE13898) confirmed that all the subtype I EAC were *TP63* positive while all the subtype II cases were *TP63* negative, *TP63* was very specific to subtype I EAC (*p* < 0.0001, Additional file 1: Table S1) [21].

## Discussion

Through meta-analysis, we identified and validated that there are two molecular distinct subtypes of EAC in three independent cohorts. Although these two subtypes had similar clinical-pathological characteristics including age, gender, grade, history of alcohol and smoking, degree of differentiation, they differed in expression profiles, mutation profiles and T-staging. Specifically, we identified TP63 was specific to subtype I EAC, suggesting a potential biomarker for further investigation and molecular analysis.

Gastrointestinal reflux has been reported to be one of the major causes for metaplasia of esophagus and BE [27]. The reflux activates the columnar differentiation pathway through gain of expression of intestinal transcription factors, including CDX1, CDX2, GATA4, GATA6, HNF1α, TGFβ. Since these genes expressed in the similar levels between the two molecular subtypes, it suggested that the columnar differentiation might be activated for both molecular subtypes by gastrointestinal reflux. Meanwhile, the reflux would also inactivate the squamous differentiation pathway with expressional loss of some critical transcription factors, including TP63 and SOX2, which lead to the squamous de-differentiation manifested by the reduced expression of squamous differentiation markers, including keratin 4, keratin 14, and SPRRs. However, these squamous differentiation related genes were found to be significantly higher expressed in subtype I EAC than subtype II EAC cases, indicating that both molecular subtypes accompanied with the columnar differentiation but EAC from different molecular subtypes underwent different degrees of squamous de-differentiation. BE was reported to be the precursor of EAC. Interestingly, our results showed that subtype II EAC, not subtype I EAC, shared more common molecular expression patterns with BE.

The landscape of somatic mutations in EAC has been widely analyzed [1, 28–31]. A number of genes were found to be significantly mutated in EAC in the earlier stages, including *TP53*, *CDKN2A*, *SMAD4* and *ARID1A* [28]. By analyzing the somatic mutations in view of the two molecular subtypes based on the TCGA dataset, we found that *TP53* and *CDKN2A* are as reported to be commonly mutated in the majority of EAC patients regardless of subtypes, indicating that *TP53* mutations may be early events in the development of EAC [1, 29]. Nevertheless, 24 and 30 genes were only significantly mutated in subtype I and subtype II EAC patients, respectively. *SMAD4* and *ARID1A* have been reported to be significantly mutated in EAC patients previously [28]. However, within our current study, *SMAD4* was only found to be significantly mutated in subtype I, in contrast, *ARID1A* was only significantly mutated in subtype II EAC patients, indicating EAC subtype-specific mutation profile and possible subtype-specific tumorigenesis mechanism. Although about two dozens of genes significantly mutated in each subtype, the cell cycle pathway was shown to be enriched in both mutated gene sets from subtype I (*TP53*, *CDKN2A*, *SMAD4*) and subtype II (*TP53*, *CDKN2A*, *RB1*, *CDC7*) EAC, indicating the important function of the cell-cycle pathway in both subtypes.

In this study, we also performed the primary analysis of chemotherapy response in EAC patients despite a limited number of patients have available therapeutic information. We showed that subtype II EAC was more sensitive to frontline chemotherapy while subtype I was more tolerant to the chemotherapy although the association between molecular subtypes and chemotherapy response did not reach significance, nevertheless it is still worth to measure the association between EAC molecular subtypes and chemotherapy in a larger sample size since the association assessment in current study was analyzed on only three patients. *NGFR*, known as nerve growth factor receptor, expressed in the basal cells of the normal esophageal epithelium and a subset of cells of ESCC, was found to be expressed higher in subtype I EAC than subtype II EAC. The NGFR+ cells in ESCC were more likely to be the cancer stem cell, and more resistant to chemotherapy drug DDP than NGFR- cells of ESCC [32]. This may also indicate the resistant roles of NGFR for the chemotherapy response in the treatment of subtype I EAC patients. In addition, NGFR was reported to be a potential biomarker for molecular subtypes of breast cancer, including basal-like breast cancer and luminal B breast cancer [33]. Future study targeting NGFR in EAC may not only help to stratify the molecular subtypes of EAC but may also help to overcome the chemotherapy resistance and develop novel targeted therapeutic methods for subtype I EAC patients. CXCR2 is a member of the G-protein-coupled receptor family and a receptor for IL8 (interleukin 8) and CXCL1. CXCL1 is a protein showing melanoma growth stimulating activity. In our study, CXCR2 was significantly over-expressed in subtype I EAC than subtype II EAC. A study targeting CXCR2 in EAC cell line demonstrated that inhibition of CXCR2 with a small molecular inhibitor (SB332235) suppressed the invasiveness of EAC derived OE33 cells [34], suggesting that subtype I EAC may exhibit high sensitivity to CXCR2 targeted therapy.

When comparing subtype I specific genes and chemo-resistant relative genes, 45 genes, including *ATP6V0A4*, *BMP7* and *KLK11*, were found to be over-expressed in both groups. *ATP6V0A4* is one subunit of v-ATPase which was reported to be involved in chemo-resistance and invasion of tumor cells [35–37] and a biomarker for specific subtypes of human gliomas [35]. Also, *ATP6V0A4* is one of the 45 genes highly expressed in subtype I and chemo-resistant EAC patients. *ATP6V1C1*, a family member of *ATP6V0A4*, was highly expressed in BE and EAC, could be blocked by esomeprazole, resulted in antineoplastic effects and inhibition of proliferation, cell invasion and apoptosis of EAC cells [38]. In addition, *ATP6V1C1* was also reported to display resistance to cytotoxic drugs [39–41], and therefore could be a potential therapeutic target for chemo-resistant tumor treatment [42–46]. *BMP7*, bone morphogenetic protein 7, was found to be consistently over-expressed in chemo-resistant ovarian cell line than chemo-sensitive ovarian cell line [47]. By analyzing gene expression of clinical primary advanced colorectal cancer, Li et al. reported that expression signature of *HOXB8* and *KLK11* could predict the effects of FOLFOX4 chemotherapy in primary advanced colorectal cancer patients [48]. The same research group also knocked down *KLK11* in colorectal cancer cell line and shown that decreasing of *KLK11* expression inhibited the cell proliferation and enhanced the sensitivity to oxaliplatin [49].

Our study suggested that subtype II EAC patients might be more likely responsive to chemo-therapy. However, because only limited clinical information, such as chemotherapy and TNM staging, from a small number of EAC patients available, this association between chemo-therapy and EAC molecular subtypes needs to be explained with caution. Given future analyses of more comprehensive clinical information from larger EAC patient populations support our findings, this subtype-specific chemo-therapy response would certainly help guide the appropriate clinical treatments for patients with different subtypes.

## Conclusions

In summary, the analysis performed in this study identified and validated the two molecular subtypes of EAC with different expression and somatic mutation patterns. Meanwhile, the subtype II EAC was shown more sensitive to the frontline chemotherapy than subtype I EAC.

## Methods

To identify and define the molecular subtypes of EAC, we collected the GEO datasets with plenty of EAC samples (≥50 cases), including GSE13898 (75 EAC cases) [21] and GSE19417 (52 EAC cases) [22]. In addition, level 3 RNA-Seq and exome-Seq data of EAC (88 cases) were collected from TCGA database as well at March 17, 2015 [50]. After filtering expression profile to genes by a standard deviation (SD), the Consensus Clustering (R package ConsensusClusteringPlus) [51] for each of the three expression datasets was performed to determine the optimal number of subtypes and to assign the subtype for each EAC case, Consensus Clustering resampled samples/genes with ratio 80% and executed agglomerative hierarchical clustering, this process was ran over for 1,000 iterations as performed previously [3, 4, 52]. The statistical significance of subtypes in each dataset was examined by SigClust analysis [53]. Silhouette analysis (R package cluster) [54] was then used to measure the accuracy of subtype assignment from ConsensusClusteringPlus. Subclass mapping was used to determine the reproducibility of molecular subtypes between three above expression profiling datasets [55]. Subtype specific gene expression patterns and pathways were analyzed by Gene Set Enrichment Analysis (GSEA) [56], DAVID Bioinformatics Resources [57, 58] and Significance Analysis of Microarrays (SAMseq) [59]. Cluster 3.0 was used to do hierarchical clustering [60] and clustering result was viewed by TreeView [61]. The significantly mutated genes were identified in entire EAC cohort or in subtype specific manner by MutSigCV analysis [62]. The contingency analyses performed in this study were assessed by the chi-square and Fisher exact tests using GraphPad Prism software and *p* value less than 0.05 was considered significant.

## Additional files

Additional file 1: **Table S1.** Clinical features of two EAC molecular subtypes. **Table S2.** Differentially expressed genes between two EAC molecular subtypes by SAMseq. **Table S3.** Biological processes annotation of Gene Ontology for differentially expressed genes from SAMseq. **Table S4.** KEGG Pathway annotation for differentially expressed genes from SAMseq. **Table S5.** Overlapped genes between subtype I specific genes and chemo-resistant genes. **Table S6.** The mean number of mutations per case in different EAC subtypes by TCGA exome-seq data. (XLSX 94 kb)

Additional file 2: **Figure S1.** The number of mutations per EAC case from different EAC subtypes. (TIFF 98 kb)

## Abbreviations

BASAL set: HUPER_BREAST_BASAL_VS_LUMINAL_UP
BE: Barret’s esophagus
EAC: Esophageal adenocarcinoma
EC: Esophageal carcinoma
ESCC: Esophageal squamous carcinoma
GSEA: Gene set enrichment analysis
WANG set: WANG_BARRETTS_ESOPHAGUS_AND_ESOPHAGUS_CANCER_DN
