# Supplementary figures and images for: Distinct esophageal adenocarcinoma molecular subtype has subtype-specific gene expression and mutation patterns
Source: BMC Genomics. 2018 Oct 24;19:769. doi: 10.1186/s12864-018-5165-0 (PMC6201634; doi:10.1186/s12864-018-5165-0)

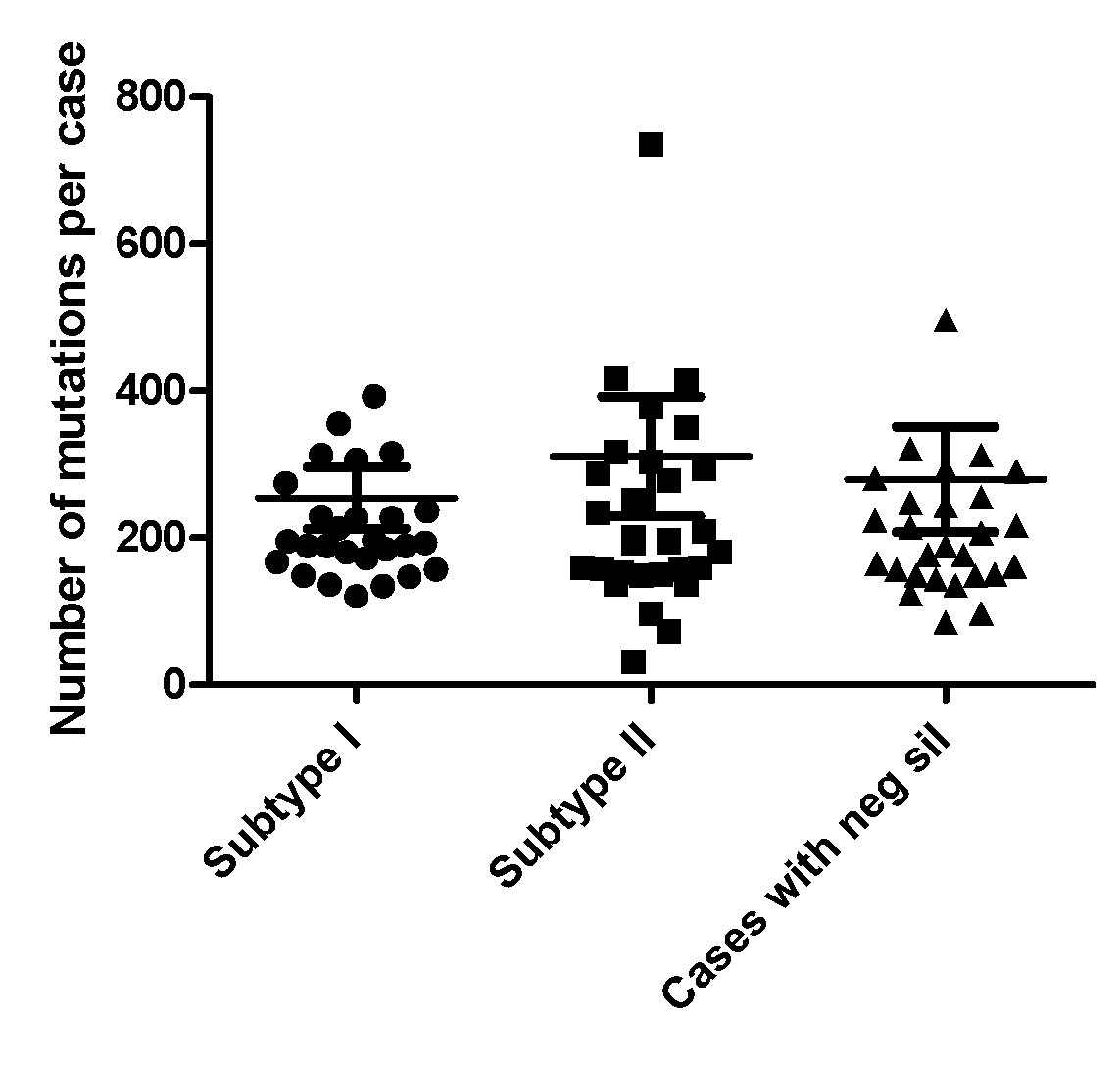

Supplement: Supplementary file 2 — Figure S1. The number of mutations per EAC case from different EAC subtypes. (TIFF 98 kb) [file 12864_2018_5165_MOESM2_ESM.tiff]
